# Therapeutic drug monitoring guides personalized ponatinib dose reduction for chronic myeloid leukemia

**DOI:** 10.1007/s12185-026-04254-7

**Published:** 2026-07-14

**Authors:** Maiko Abumiya, Naoto Takahashi

**Affiliations:** 1 Department of Pharmacy, Akita University Hospital, Akita, Japan; 2 Department of Hematology, Nephrology and Rheumatology, Akita University Graduate School of Medicine, 1-1-1 Hondo, Akita City, Akita 010-8543 Japan

**Keywords:** Chronic myeloid leukemia, Ponatinib, Therapeutic drug monitoring, OPTIC trial, Arterial occlusive events

## Abstract

The arterial occlusive events (AOEs) associated with ponatinib are dose-dependent. Although the phase II OPTIC trial showed that reducing the dose to 15 mg daily (QD) after achieving a response provided an optimal benefit-risk profile, this has not been adequately verified using plasma drug concentrations data. A 66-year-old woman with chronic-phase chronic myeloid leukemia (CP-CML) was switched to ponatinib because of nilotinib-induced grade 3 thrombocytopenia. For safety, ponatinib was initiated at 15 mg every other day (Q2D) and gradually increased to 45 mg QD. A major molecular response (MMR) was achieved 30.6 months after starting ponatinib. The dose was then reduced to 15 mg QD to mitigate cardiovascular risks, and the MMR was successfully sustained for approximately 2 years (21.2 months) at this reduced dose. The total overall observation period was more than 5 years (65.2 months). A strong correlation was observed between the ponatinib dose and plasma trough concentrations (R^2^ = 0.8132). Although 30 mg QD was required to reach the target concentration for suppressing resistant clones (23 ng/mL), MMR was sustained at 15 mg QD, with a trough level of 14.1 ng/mL. Once the tumor burden is significantly reduced, lower concentrations of ponatinib may be sufficient to maintain the molecular response.

## ntroduction

Cardiovascular events, particularly arterial occlusive events (AOEs), are among the most serious adverse effects of ponatinib therapy in treatment-resistant chronic-phase chronic myeloid leukemia (CP-CML). These events are known to be dose-dependent, making dose optimization a critical strategy for balancing efficacy and long-term cardiovascular safety.

Although the international Phase II OPTIC trial demonstrated that starting at 45 mg QD and reducing to 15 mg after achieving the BCR::ABL1 International Scale transcript (BCR::ABL1^IS^) ≤ 1% significantly reduces AOE risks while maintaining efficacy [1], real-world application requires careful monitoring. Individual pharmacokinetic variability can lead to significantly different systemic exposures even at identical doses.

Previous research proposed standardizing ponatinib therapeutic drug monitoring (TDM) in Japanese patients, suggesting a target plasma trough concentration of 23 ng/mL to adequately suppress resistant clones [2]. However, longitudinal evaluations of plasma drug concentrations during the OPTIC-style dose-reduction phase remain extremely limited in clinical practice.

Herein, we report a case of CP-CML in which individualized dose adjustments and a subsequent safe ponatinib reduction were successfully guided by longitudinal TDM. This case demonstrates the clinical utility of measuring trough concentrations to support personalized safety strategies without losing molecular response.

## Case report

In September 2019, a 66-year-old woman was diagnosed with BCR::ABL1-positive CP-CML (Sokal score: high risk). She was non-smoker with a BMI of 24.0 and no history of diabetes. First-line nilotinib therapy 300 mg twice a day (BID) was initiated on September 11, 2019. However, due to grade 3 thrombocytopenia, nilotinib was discontinued after 7.3 months, and the treatment was switched to ponatinib on April 22, 2020. At that time, the BCR::ABL1 ^IS^ was 11.8%, indicating treatment failure according to the ELN 2020 criteria. We performed mutation analysis using the direct sequencing method, but no resistance mutations, including T315I, were detected (with a detection limit of approximately 15–20% variant allele fraction).

Owing to persistent grade 3 thrombocytopenia, ponatinib was initiated at 15 mg every other day (Q2D) to prioritize safety while searching for an effective dose. The patient received 15 mg Q2D for the first 5.5 months, after which the dose was escalated to 15 mg QD for 5.8 months, 30 mg QD for 10.5 months, and finally reached 45 mg QD at 21.8 months after ponatinib initiation. The patient successfully achieved an MMR (BCR::ABL1 IS ≤ 0.1%) on November 8, 2022, which was 30.6 months after starting ponatinib. The platelet counts normalized within 4 months after switching to ponatinib. Although she developed hypertension and dyslipidemia during ponatinib treatment, these conditions were well managed with amlodipine, telmisartan, and simvastatin.

**Fig. 1:**
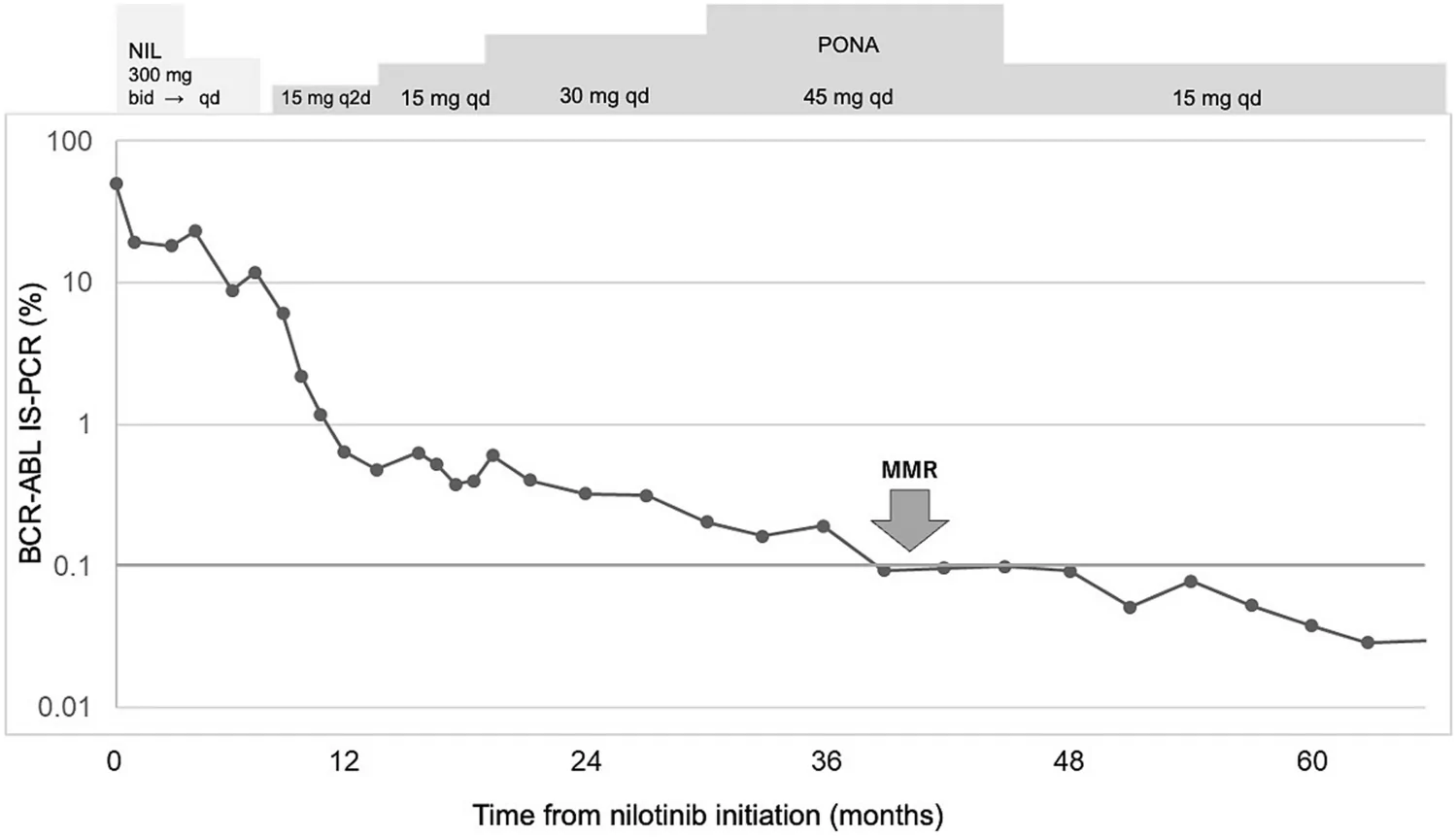
Clinical course and transitions in BCR::ABL1 transcript levels. Escalation phase: Ponatinib was initiated at a low dose of 15 mg every other day (Q2D) to ensure safety and gradually escalated to 45 mg daily (QD). Reduction phase: A major molecular response (MMR, BCR::ABL1^IS^ ≤ 0.1%) was achieved 30.6 months after starting ponatinib. Subsequently, the dose was reduced to 15 mg QD to mitigate the risk of arterial occlusive events (AOEs). The MMR was successfully maintained despite the dose reduction. The total overall observation period (including prior nilotinib therapy) was 65.2 months. Time zero on the x-axis corresponds to the initiation of nilotinib therapy (ponatinib was initiated at month 7)

To minimize the risk of long-term cardiovascular events, the dose was reduced to 15 mg QD on May 9, 2023, after maintaining a stable MMR at 45 mg QD for 6.0 months. The MMR was successfully sustained for 21.2 months (approximately 1.8 years) of follow-up at the 15 mg QD dose, up to the last follow-up on February 15, 2025. The total duration of ponatinib therapy was 57.8 months (approximately 4.8 years), and the total overall observation period (including prior nilotinib therapy) was 65.2 months (> 5 years) with no significant AOEs (Fig. 1).

## Plasma ponatinib concentrations

**Fig. 2:**
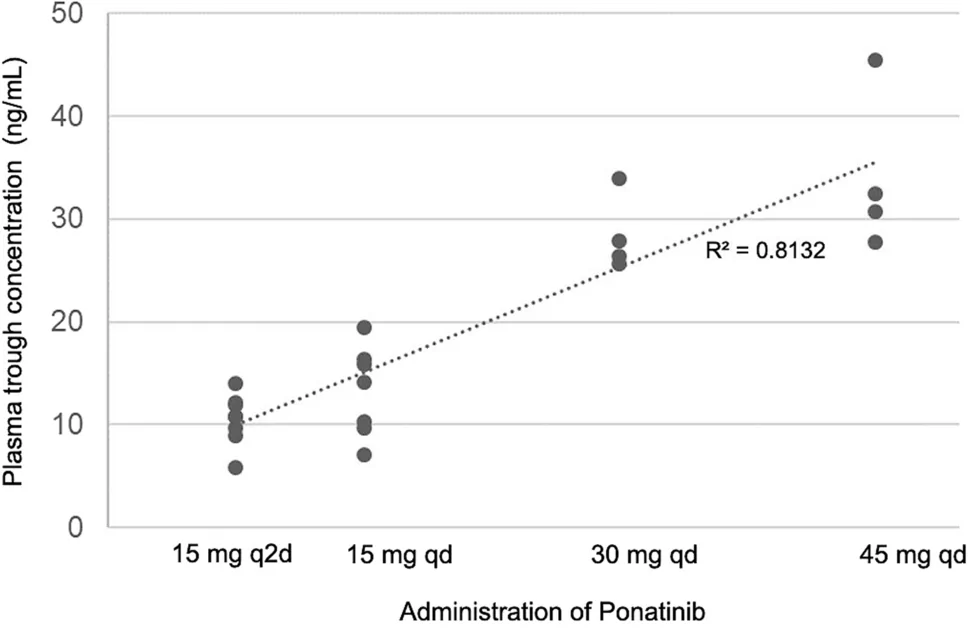
Correlation between ponatinib dose and plasma trough concentrations. Scatter plot illustrating the plasma trough concentrations at various ponatinib doses (15 mg Q2D, 15 mg QD, 30 mg QD, and 45 mg QD). The median concentrations (and coefficients of variation) were 10.8 ng/mL (23.5%, *n* = 6), 14.1 ng/mL (33.1%, *n* = 7), 27.2 ng/mL (10.8%, *n* = 4), and 30.7 ng/mL (23.0%, *n* = 4), respectively. A strong linear correlation was observed between the dose and trough concentration with a coefficient of determination (R^2^) of 0.8132. Although a dose of ≥ 30 mg QD was required to reach the previously proposed target concentration of 23 ng/mL for suppressing resistant clones, the molecular response was maintained at 15 mg QD even with a lower trough level

Plasma trough concentrations at various doses (15 mg Q2D, 15, 30, and 45 mg QD) were monitored (Fig. 2). The median trough concentrations were 10.8, 14.1, 27.2, and 30.7 ng/mL, respectively. A strong linear correlation was observed between dose and trough concentrations (R^2^ = 0.8132). It should be noted that this regression analysis plots repeated measurements from a single case (*n* = 21) and does not represent independent data points. Regarding the high coefficient of variation (33.1%) observed in the 15 mg QD group, while no direct interactions with concomitant medications (amlodipine, telmisartan and simvastatin) are known, the influence of meal timing or compliance cannot be ruled out. Furthermore, the 15 mg Q2D data were included in the analysis as measured trough levels, taking the dosing interval into account.

## Discussion

Cardiovascular events, specifically AOEs, remain the most critical concern during ponatinib therapy for CP-CML [3]. Previous multivariate analyses have established a clear dose-dependency, indicating that the risk of AOEs decreases by approximately 33% for every 15 mg reduction in ponatinib dose [4]. Therefore, identifying the minimum effective dose for each patient is vital for balancing long-term cardiovascular safety with the maintenance of molecular response.

The international Phase II OPTIC trial revolutionized the dosing paradigm for ponatinib by demonstrating that a response-based dose reduction strategy (starting at 45 mg and reducing to 15 mg upon achieving BCR::ABL1^IS^ ≤ 1%) provided an optimal benefit-risk profile [1]. Although “the OPTIC regimen” officially advocates a high-dose start, real-world data from Japan suggest that many clinicians often initiate therapy at 15 mg to prioritize safety, particularly in patients with preexisting comorbidities or histories of severe hematological toxicity [5]. Our case exemplifies this “safety-first” approach, and because of the patient’s history of nilotinib-induced grade 3 thrombocytopenia, ponatinib was initiated at 15 mg Q2D. This individualized titration allowed the recovery of platelet counts while gradually identifying the dose required to achieve MMR.

A highlight of this report is the longitudinal verification of the OPTIC strategy using TDM. We observed a strong linear correlation between the ponatinib dose and plasma trough concentration (R^2^ = 0.8132). While these measurements (*n* = 21) are longitudinal observations from a single subject, the robust R^2^ value indicates high intra-patient dose-proportionality. This correlation underscores the fact that dose adjustments directly translate into predictable changes in systemic exposure in this patient. Interestingly, our pharmacological analysis revealed that a dose of at least 30 mg QD is necessary to reach the previously proposed target concentration of 23 ng/mL. This target was established in vitro as the threshold required to suppress highly resistant clones such as those harboring the T315I mutation [2].

However, despite the trough level at 15 mg QD (14.1 ng/mL) falling below this 23 ng/mL threshold, the patient has successfully maintained MMR for several years. In CP-CML patients without detectable resistance mutations, an OPTIC-style dose reduction to 15 mg QD may be sufficient to maintain MMR even at trough concentrations below the threshold established for T315I clone suppression. This discrepancy suggests that once a major response is achieved and the total tumor burden is significantly reduced, the concentration required to suppress the remaining sensitive clones may be substantially lower than the initial “induction” threshold required for resistant disease. In this case, the absence of T315I or other major resistance mutations at the detection limit of direct sequencing likely allowed for stable disease control at a lower systemic exposure.

We propose, as a hypothesis requiring further validation, that in patients without TKI-resistant mutations, achieving a deep molecular response provides a “pharmacological buffer” where lower doses and lower systemic exposures are sufficient to prevent clonal expansion, thereby enabling individualized dose reduction to mitigate cardiovascular risks without losing efficacy.

In conclusion, the present case provides preliminary observational support for the hypothesis that TDM may assist in guiding individualized dose reduction decisions in ponatinib therapy, and prospective studies are warranted to validate this approach.

## Data Availability

Data will be made available on reasonable request.
